# Malaria Parasite Density as a Predictor of Hematological Parameter Changes among HIV Infected Adults Attending Two Antiretroviral Treatment Clinics in Kano, Northwest Nigeria

**DOI:** 10.1155/2020/3210585

**Published:** 2020-04-27

**Authors:** Feyisayo E. Jegede, Tinuade I. Oyeyi, Surajudeen A. Abdulrahman, Henry A. Mbah

**Affiliations:** ^1^Family Health International-360, Plot 1073-A1 GODAB Plaza, Area 3 Garki-Abuja, Abuja, Nigeria; ^2^Biological Science Department, Bayero University Kano, Kano, Nigeria; ^3^Health Education England, East of England, 2-4 Victoria House, Capital Park, Fulbourn, Cambridge CB21 5XB, UK; ^4^LabTrail, Global LLC, Smyrna, DE, USA

## Abstract

**Background:**

Despite public health significance of dual infections of human immunodeficiency virus (HIV) and malaria in developing countries like Nigeria, information on the association between malaria parasite density count (MPDC) and hematological parameter changes among HIV-infected individuals is rarely available.

**Objectives:**

To evaluate burden of HIV and malaria dual infections and assess the predictive association of MPDC with hematological parameter changes among HIV infected adults attending two antiretroviral treatment clinics in Kano, Nigeria. *Methodology*. This was a cross-sectional study consisting of 1521 consented participants randomly selected between June 2015 and May 2016. Participants' basic characteristics and clinical details were collected using a pretested and validated standardized questionnaire. Collected venous blood was analyzed for malaria by rapid testing and microscopy including malaria parasite density; hematological parameters were estimated using a Sysmex XP-300 autoanalyzer. Data was reviewed, cleaned, and analyzed using SPSS software version 23.0. Mean hematological parameters and HIV/malaria status were compared using the independent *t*-test; hematological parameters and MPDC relationship was tested by simple linear regression analysis. Statistically significant difference at probability of <0.05 was considered for all variables.

**Results:**

The majority (70.6%) of the participants were females. Mean (SD) age was 37.30 ± (10.41) years and ranged from 18 to 78 years. 25.4% of participants had dual infection, 99% due to *Plasmodium falciparum* species. Mean MPDC was 265 ± 31.8 (SD) cells/*μ*l and ranged from 20 to 2500 cells/*μ*l. Dual infection was highest (37.5%) among respondents in the age group ≥60 years. Prevalence was similar among other age groups (*p* = 0.165) and gender (*p* = 0.942). Of the 16 hematological parameters evaluated, 11 showed significant difference between HIV mono-infected and dual infected participants. Of the 11 parameters, only 7 (Hb, MCHC, red cells count, neutrophil and lymphocyte percentage, absolute lymphocyte count, and red cell distribution width) were significantly predictive of changes with respect to MPDC.

**Conclusions:**

MPDC was significantly predictive of changes in 7 hematological parameters among dual infected participants in these settings. In routine malaria diagnosis, MPDC determination with respect to changes in some hematological parameters should be considered in ART programs for improved patient management.

## 1. Introduction

Human immunodeficiency virus (HIV) as dual infection with malaria may possibly facilitate geographical expansion of malaria especially in areas where HIV burden is high. Additionally, transient and repeated increases in HIV viral load arise as a result of repeated episodes of malaria in dual infections and may also be an important factor facilitating the spread of HIV in sub-Saharan Africa [1, 2]. The exact nature of the relationship between HIV and malaria infection continues to be a subject of controversy. People living with HIV are at increased risk of clinical malaria which worsens over time as the immune system is suppressed, thus increasing the risk of malaria treatment failure [2]. Despite some reports that HIV does not have effect on malaria infection, malaria parasite density has been found to be significantly higher among HIV infected compared to HIV negative individuals [3]. The major challenges faced by people living with HIV are opportunistic infections like malaria and a range of other pathogenic infections, resulting in significant mortality [4]. In conformity with World Health Organization (WHO) recommendations on antiretroviral therapy (ART) program in developing countries, cotrimoxazole (CTX) prophylaxis is now an integral part of standard ART program [5]. This remains a major and important strategy for preventing most opportunistic infections among HIV infected individuals, including malaria infection [6]. In addition to CTX prophylaxis, some ART regimens [7] and insecticide-treated bed nets [2] have been reported to reduce the risk of malaria among HIV infected individuals. Unsurprisingly, discontinuation of CTX among HIV infected adults receiving ART has resulted in progressive increases in malaria burden and parasitemia [8]. Data evidence suggests that ART boosts immunity and reduces the incidence of malaria among HIV infected compared to HIV infected non-ART individuals; hence, it may possibly have indirect implication in malaria control [9]. Of particular importance among such ART regimens are protease inhibitors which have been reported to have antimalaria effects [10, 11] and have been shown to have potential ability to reduce malaria episode and prevalence [12].

HIV and malaria dual infections, particularly *Plasmodium falciparum* species, are both pathogens that induce significant perturbation and activation of the immune system. Such dual infections have been reported as the two most important health problems of developing countries, including Nigeria, accounting for more than 4 million annual deaths globally [13].

Studies have also provided evidence that anemia is the most commonly encountered hematological abnormality in HIV and malaria dual infected individuals [14–18]. However, other authors have reported different findings [19, 20]. Some studies have reported on the association between malaria and its effect on very limited hematological parameters among HIV and malaria dual infected patients without information on malaria density count [21–24]. In most sub-Saharan African countries, where the burden of HIV and malaria dual infections is incidentally high, comprehensive evaluation of hematological parameters should be considered. Thus far, only one study in Jos, Nigeria, has evaluated the effect of malaria parasite density on hemoglobin level only among HIV-infected population [25]. The major objective of this study was to determine the predictive association of malaria parasite density with hematological parameter changes among HIV infected adults attending two antiretroviral treatment clinics in Kano, Nigeria.

## 2. Methodology

### 2.1. Settings

This study was conducted between June 2015 and May 2016 in two public secondary health facilities, namely, Infectious Diseases Hospital (IDH) Kano and Murtala Muhammad Specialist Hospital (MMSH) Kano in Northwest Nigeria. The two facilities were purposefully selected because, first, IDH was the first public secondary health facility to commence ART services in Kano state followed by MMSH. Second, both facilities have high patient volume and presumed availability of previous patients' information and data in both electronic management system and individual patient folders. Third, necessary ART laboratory equipment to support monitoring of patients was available.

#### 2.1.1. Infectious Disease Hospital Kano

IDH Kano is located at France road, Sabon Gari, Fagge local government area of Kano state (Latitude 12.0113828′N and longitude 8.530433′E) within the savanna. The facility was established in the early 1960s as an isolation unit for smallpox patients. Subsequently, the facility was expanded into a fully fledged secondary level state-owned public hospital which now caters for all epidemic diseases prevalent in the state. The hospital provides services to a catchment area around Kano and neighboring states as well as to patients from the Republic of Niger. Presently, the hospital has a capacity of about 250 beds and a new multidrug resistant (MDR) tuberculosis clinic with three additional wards. With funds from the President's Emergency Plan for Aids Relief (PEPFAR) through the United States Agency for International Development (USAID), Family Health International (FHI-360) in collaboration with Kano state government has been providing comprehensive ART program support to the facility since February 2005 till date. The ART laboratory provides HIV serological screening and ART monitoring testing as well as other tests such as pregnancy tests, hepatitis B and C, syphilis, and microscopy for malaria and tuberculosis diagnosis. Since inception, a total of about 7,000 HIV-positive patients are currently receiving ART as of July 2019.

#### 2.1.2. Murtala Muhammad Specialist Hospital Kano

MMSH Kano is located at old Kano city metropolis (latitude 11.998190′N and longitude 8.522948′E) in Northwest Nigeria within the savanna. MMSH started service in 1926, and presently, the hospital has over 500 beds with an average of about 6000 outpatient encounters daily based on records. Through similar funding mechanism to that of IDH, comprehensive ART program support was started since August 2006. The ART laboratory provides similar services to those of IDH Kano. Since inception, a total of about 5,000 HIV positive patients are receiving ART as of July 2019.

### 2.2. Ethical Approval and Consent to Participate

The research protocol was reviewed and approved by Kano State Hospitals Management Board Ethical Committee (REF: HMB/GEN/488/1 of 17^th^ April 2015). All respondents voluntarily participated in the study, and signed individual consent forms before enrollment into the study.

### 2.3. Study Design, Eligibility, and Sample Size Determination

This was a cross-sectional study involving (randomly selected) consenting HIV-infected adults of both sexes aged 18 years and more and attending HIV treatment and care clinics of IDH Kano and MMSH Kano. We estimated the sample size with STATA software using formula for hypothesis testing of two-mean comparison. The estimates were based on assumptions of similar studies data in Nigeria [20, 26] and considered outcomes variables for our calculation (hemoglobin concentration, red blood cells, and platelets count). To determine and obtain an effect size of 1.5 × 10^12^/l for the red blood cells, a total of 200 participants per group was required to provide an 80% power in detecting this difference, assuming a two-tailed test and significance level of 5%. Details of sample size analysis were reported previously [27]. Of all outcomes, RBC yielded the largest sample size and hence was adopted for this study. Further adjustment of 5% nonresponse was made, and we eventually enrolled 761 participants from IDH and 786 participants from MMSH. A total of 26 participants with incomplete details were excluded giving a total of 1521 participants for the study.

### 2.4. Inclusion and Exclusion Criteria

Participants that visit Infection Disease Hospital Kano and Murtala Muhammad Specialist Hospital Kano to access antiretroviral services were approached and recruited into the study, having satisfied the following inclusion criteria:

- Adults aged 18 years and above
- Confirmed HIV infection
- Duly registered into the antiretroviral treatment (ART) program at both hospitals

### 2.5. Exclusion Criteria

Exclusion criteria include the following:

- Pregnant women
- Any HIV-infected participant who has been on antimalarial drugs in the past two weeks
- All those who did not consent to participate

### 2.6. Malaria Diagnosis, Parasite Density Determination, and Hematological Parameter Estimation

In brief, malaria parasites were screened, using malaria rapid diagnostic test (RDT) kits as described by the manufacturers (Standard Diagnostics Bioline, 2013). Regardless of RDT results, the blood smear microscopy method was used for confirmation. Blood sample collection, malaria diagnosis, and hematological parameters determination procedures had been previously described [27]. Malaria parasite density was determined more accurately using the actual white blood cells count per microliter, unlike the assumed white blood cells count of 8000 WBC/*μ*l estimation recommended by the WHO [28]:

(1) $$\begin{array}{c} \text{malaria parasite density estimation }=\frac{\left(\text{number of parasite count }\times \text{ patient actual white blood cell count}\right)/µ\text{l}}{\text{number of white blood cell count }200\;\mathrm{or}\;500}. \end{array}$$

Hematological parameters and platelets were estimated using an automated Sysmex XP-300 analyzer. To produce quality results with precision and accuracy, daily analysis of EIGHTCHECK control stabilized blood consisting of low, medium, and high levels of controls was done according to manufacturer instruction [29].

### 2.7. Data Collection and Statistical Analysis

All data collected were entered into Microsoft Excel files and reviewed for correctness, completeness, and consistency. Data confidentiality was ensured through delinking participant names from serial and enrollment number and restricting data access to only authorized research team members.

Cleaned data were imported into Statistical Package for Social Sciences (SPSS) software version 23.0 for analysis. Univariate analysis which includes descriptive statistics like frequencies, percentages, and exploration of the distribution of all variables of interest was performed. Association between categorical variables was tested using the chi-square test, i.e., association between HIV and malaria dual infected status in relation to age and gender. The mean values of hematological parameters by HIV/malaria dual infected status were generated and compared using the independent sample *t*-test. The association of MPDC with hematological parameters that showed statistically significant difference in the initial analysis was further tested using simple linear regression analysis. All tests were two-tailed with a significance set at 5% (0.05).

## 3. Results

### 3.1. Basic Characteristics and Clinical Information of Studied Participants

A total of 1521 HIV-positive individuals consisting of 1074 (70.6%) females and 447 (29.4%) males with a mean and standard deviation of 37.20 ± 10.41 and age range of 18–78 years were evaluated. Majority (79.8%) were of Hausa/Fulani ethnicity with 85% residing in urban settings. Greater proportion (71.3%) of the studied population had completed some level of education from primary to tertiary levels (Table 1). A major proportion (45.9%) of the studied participants were self-employed while about 12% were civil servants and about 19% were unemployed at the time of this study. More than half (57.1%) of the study population were married, followed by approximately 23% who had lost their spouses. Over three quarters (79.2%) of the participants did not complain about their health at the time of visit during this study. Among those who had health complaints, about 38% complained of fever/headache/others combined, while about 32% complained of body pain/body weakness/others (Table 1). About 74% of participants reported using insecticide treated mosquito bed nets. Of those who use the treated bed nets, majority (80.3%) used them consistently for seven days per week (Table 1).  Table 2 shows clinical information such as ART status, WHO staging, and CTX daily use.

### 3.2. Prevalence of HIV and Malaria Coinfection in Association with Sociodemographic Characteristics (Age and Gender)

In this study, the percentage of HIV and malaria dual infection was 25.4% based on the gold standard method (blood smear microscopy). Almost all (99.2%) of the HIV and malaria dual infections were due to *Plasmodium falciparum* species. Dual infections of *Plasmodium falciparum* and *Plasmodium vivax* species was observed in only 3 participants (0.8%). Analysis based on age group revealed that a majority (35.7%) of HIV and malaria dual infected individuals were in the 60-year and above age groups. Prevalence of HIV and malaria dual infection was similar in both genders. Overall, age group and gender did not show a statistically significant difference in prevalence of HIV and malaria dual infection (Table 3).

### 3.3. Pattern of Hematological Parameters and Its Association with Malaria Parasite Density Count among HIV and Malaria Dual Infected Participants

A total of 16 hematological parameters were evaluated and compared among participants who were HIV and malaria dual infected and those who were HIV infected only. These 11 parameters showed a statistically significant mean difference between HIV infection only and HIV and malaria dual infection. The parameters were (1) packed cell volume, (2) hemoglobin concentration, (3) red blood cell, (4) mean cell volume, (5) mean cell hemoglobin concentration, (6) mean cell hemoglobin, (7) absolute lymphocyte count, (8) neutrophil differential count, (9) lymphocyte differential count, (10) mixed differential count, and (11) red cell distribution width. Eight hematological parameters ((1) packed cell volume, (2) hemoglobin concentration, (3) red blood cell, (4) mean cell hemoglobin, (5) mean cell hemoglobin concentration, (6) mean cell volume, (7) absolute lymphocyte count, and (8) red cell distribution width) showed significantly higher mean among HIV infected individuals without malaria (Table 4).

Furthermore, we estimated malaria parasite density count (MPDC) and its association with hematological parameters among the HIV and malaria dual infected participants. Mean MPDC was 265 ± 31.8 (SD) cells/*μ*l with a range of 20 to 2500 cells/*μ*l (Table 3). Subsequent analysis of 11 blood count parameters showed significant difference among the HIV and malaria dual infected participants, revealing that MPDC was associated with changes in seven out of the 11 parameters, and the association was statistically significant. For every unit increase in MPDC, Hb decreased significantly by 0.98 g/dl, RBC by 0.252 × 10^12/L^, MCHC by 1.76 g/dl, lymphocytes by 5.68%, and absolute lymphocyte count by 0.083 × 10^9/L^ (Table 5). Conversely, neutrophils and RWD increased by 6.96% and 0.021, respectively, for every unit increase in MPDC. These relationships were all statistically significant (*p* < 0.05) (Table 5).

## 4. Discussion

### 4.1. Summary of Main Findings

We observed a 25% prevalence of HIV and malaria dual infections in two ART clinics across two local government areas of Kano state of Nigeria. *Plasmodium falciparum* species accounted for 99% of malaria infection, and the rest were mixed infections of *Plasmodium falciparum* and *Plasmodium vivax*. In addition, mean MPDC among HIV and malaria coinfected individuals was 265 ± (SD) 31.8 cell/*μ*l and ranged between 20 and 2,500 parasite/*μ*l.

The means of 11 hematological parameters were significantly different between HIV monoinfected and HIV and malaria dual infected participants. Among dual infected participants, MPDC was observed to be a significant predictor of changes in seven out of eleven hematological parameters evaluated.

### 4.2. Comparison with Existing Literature

#### 4.2.1. Prevalence of HIV and Malaria Dual Infection

The dual infections burden of 25% in this study was similar to that in other previous studies in Nigeria in Benue [9], Jos [30], and Kano [27] states, where the prevalence of dual infections was reported as 20.5%, 24%, and 27%, respectively. The current findings of dual infections of HIV and malaria were however lower compared to some other previous studies that reported 32.2% in Kano [31], 33.5% in Ondo [32], 47.7% in Lagos [18], 74.3% in Benin City [24], and 93.3% in Port Harcourt [26] all in Nigeria. Conversely, observed dual infections prevalence in this study was higher than reports of 2.9% in Lagos [18], 7.2% in Kogi [33], and 18.9% in southeast Nigeria [34]. As of the time of this study, none of our participants were on antimalaria drugs. Notably, those on antimalaria therapy in the past two weeks were under one of the study exclusion criteria.

Compared to other African countries, we observed a higher dual infection prevalence than 15.5% and 11.7% reported in two studies in Ghana [17, 35], 10% in South Africa [36], 17.1% in Ethiopia [37], and 19.9% pooled prevalence from meta-analysis of 23 studies across Africa [14]. Elsewhere, higher dual infections prevalence of 61.7% and 33% was reported in two separate studies in Mozambique [15, 38]. Recent comprehensive and more convincing data from a systematic review reported a 0.7%–47.5% prevalence of HIV and malaria dual infections among nonpregnant and adult population across sub-Saharan Africa [39]. Arguably, we believe that wide range and variation in prevalence of HIV and malaria dual infections may be multifactorial in nature. Factors such as number of participants examined, immunity and viral load of participants, frequency of malaria exposure, study design, period of study (raining or dry season), and ratio of non-ART and ART naïve participants may influence results among different populations.

#### 4.2.2. Association of HIV and Malaria Dual Infection with Age and Gender

Prevalence of HIV and malaria dual infected burden analysis in association with gender revealed similar pattern in both sexes with no statistically significant difference. This was consistent with gender distribution data in studies conducted in Myanmar [40] and Mozambique [38] and other previous studies conducted across different states in Nigeria [9, 16, 24, 27]. However, the above observations varied with findings of Akinbo et al. who reported a statistically significant higher dual infection prevalence among male participants compared to their female counterparts in Kogi state, Nigeria [33].

HIV and malaria dual infections prevalence showed similar pattern among most age groups except those aged 60 years and more that revealed higher dual infections. Irrespective of this observation, overall, there was no statistically significant difference in dual infections prevalence observed across various age groups as previously reported in IDH Kano [27]. In contrast, significantly higher dual infections among age group of 19–24 years were observed in Kogi state, Nigeria [33]. Another study in Mozambique reported that younger age group (40 years and below) could be a significant predictor of malaria among HIV dual infected individuals [38]. Evidence from recent systematic review has provided more insight; that is, malaria and HIV dual infections is associated with an increased occurrence of clinical parasitemia, severe malaria, high parasite density, and viral load, which in turn negatively impact immunity among various groups of patients [39].

#### 4.2.3. Differences in Mean Hematological Parameters between Participants with HIV and Malaria Dual Infection and Those with HIV Monoinfection

Out of the 16 hematological parameters evaluated, 11 showed statistically significant mean difference between HIV and malaria dual infected and HIV monoinfected participants. Of those 11 hematological parameters, higher mean values were observed in seven parameters among HIV mono-infected compared to dual infected participants (Table 3). Results of the current study varied from findings by Erhabor and colleagues [26] who observed significantly higher pancytopenia among parasitized participants compared to nonparasitized control group due to the effects of dual infections. However, our findings were similar to an Ethiopia observation that revealed higher prevalence of anemia and thrombocytopenia among dual infected individuals compared to HIV negative individuals with malaria parasitemia and those without malaria parasitemia [16]. Furthermore, a study conducted in Myanmar observed that HIV and malaria coinfections significantly lower hemoglobin concentration, suggesting the possibility that coinfections may increase malaria density, further worsening malaria disease severity [40].

#### 4.2.4. Association between Malaria Parasite Density Count and Hematological Parameters among Participants with HIV and Malaria Dual Infection

With simple linear regression analysis, we demonstrated that MPDC was a significant predictor of changes in seven (Table 5) out of the 11 (Table 4) hematological parameters that were shown in our preliminary analyses to be significantly different between HIV monoinfected and HIV and malaria coinfected patients. Aside from well reported side effects of granulocytopenia and anemia associated with ZDV-containing ART regimen, anemia among HIV infected individuals has been shown to be more broadly multifactorial in origin, and three basic pathophysiologic mechanisms of anemia in HIV infection have been postulated [41]: decreased RBC production, increased RBC destruction, and ineffective RBC production. To this extent, attribution of cause and effect association of anemia in HIV infection to specific ART regimen alone may not be plausible; therefore continuous evaluation to ascertain specific cause of anemia among HIV infected individuals is critical while also ensuring that adequate monitoring and interventions are put in place [42]. Besides hematological parameters, we had provided evidence that there exists statistically significant (*p* < 0.05) variation in MPDC in relation to ART regimen, cotrimoxazole prophylaxis status, fever status, and CD+4 count [43]. The overall mean white blood cells count of 5.99 ± 2.40 cells × 10^9/l^ observed in this study was similar to 5.5 cells × 10^9/l^ reported among general population in Brazil—a malaria-endemic country [44]. Unexpectedly, the reference interval value for WBC count among Nigerians was lower (male 4.40 ± 0.86, female 4.61 ± 1.05 × 10^9/l^) [45] compared to HIV monoinfected participants in our study. In addition, low WBC count among the general population has been observed in two malaria-endemic zones [44, 45]. The risk of overestimating MPDC may arise from the use of the assumed mean WBC count of 8.0 × 10^9/l^ recommended by the WHO [28]. Such claims should trigger the need for further studies across Nigeria and elsewhere to ascertain facts. Regional and demographic variables may have contributed to such variation between assumed WHO mean WBC count and the actual individual values.

### 4.3. Strengths and Limitations of the Study

The strengths of this study include the large sample size across two local government areas, which gave the study enough statistical power and potential for generalization of the study findings among HIV positive participants this setting. In addition, the actual mean white blood cell count for each individual was determined and used for MPDC estimation instead of the assumed WHO value [28]. Thus, MPDC estimation in this study can be considered relatively reliable.

Notably, the hematology analyzer Sysmex XP-300 available at the two laboratories for routine hematology work was limited because it is a three-part differential count capacity autoanalyzer (neutrophils, lymphocytes, and mixed population) unlike a five-part differential count autoanalyzer. Therefore, mixed white blood cell types consisting of monocyte, basophils, and eosinophils cells were reported as mixed white blood cell population. A total of 51 data items were excluded in the final analysis because of incomplete information both in the clinical and the laboratory area.

We recommend further studies such as prospective cohort design with focus on HIV and malaria dual infection interaction, as well as impact of viral load, hematological parameters, and MPDC to provide more information on effect of HIV and malaria dual infection in endemic areas. Based on the distribution of WBC count among HIV participants, MPDC should be estimated and considered as an integral component of ART programs in malaria-endemic settings.

## 5. Conclusion

Dual infection of HIV and malaria burden was 25% among studied participants with no significant difference by age group and gender. Eleven blood cell count parameters were significantly different between HIV and malaria dual infected individuals and HIV monoinfected individuals. MPDC was a significant predictor of changes in seven blood cell count parameters. Diagnosing malaria, especially in advanced HIV infection including MPDC determination to monitor possible changes in hematological parameters, should be considered as an integral part of ART program in endemic settings.

## Figures and Tables

**Table 1:** Baseline characteristics of HIV-infected participants attending two selected ART clinics in Kano from June 2015 to May 2016 (*n* = 1521).

Variable	Number (%)
Gender	
Male	447 (29.4)
Female	1074 (70.6)
Age group (years)	
Less than 30	331 (21.8)
30–39	596 (39.2)
40–49	377 (24.8)
50–59	161 (10.6)
60 and above	56 (3.7)
Place of residence	
Rural	227 (14.9)
Urban	1294 (85.1)
Marital status	
Married	868 (57.1)
Single	194 (12.8)
Divorced	112 (7.4)
Widow/widower	347 (22.8)
Educational status	
No formal education	451 (29.7)
Some primary education	80 (5.3)
Completed primary	295 (19.4)
Completed secondary	518 (34.1)
Completed tertiary	177 (11.6)
Ethnicity	
Hausa/Fulani	1214 (79.8)
Yoruba	31 (2.0)
Igbo	52 (3.4)
Others	224 (14.7)
Complaints reported	
No	1204 (79.2)
Yes	317 (20.8)
Type of complaints	
Fever/headache/others	120 (37.9)
Body pain/weakness/others	100 (31.6)
Stomach upset/others	50 (15.8)
Body rashes/chest pain/stooling	25 (7.9)
Vomiting/others	22 (6.9)
Treated bed nets usage	
No	399 (26.2)
Yes	1122 (73.8)
Frequency of net usage/week	
1-2 days/week	24 (2.1)
3-4 days/week	129 (11.5)
5-6 days/week	67 (6.0)
7 days/week	902 (80.3)
Occupation	
Civil servant	186 (12.2)
Farmer	35 (2.3)
Business	515 (33.9)
Artisan	24 (1.6.0)
Student	56 (3.7)
Housewife	320 (21.0)
Unemployed	285 (18.7)
Others	100 (6.6)

**Table 2:** Clinical information of HIV-infected participants attending two selected ART clinics in Kano from June 2015 to May 2016 (*n* = 1521).

Variables	Number (%)
ART status	
ART	1288 (84.7)
Non-ART	233 (15.3)
ART start year	
2005	54 (3.6)
2006	34 (2.2)
2007	130 (8.5)
2008	156 (10.3)
2009	155 (10.2)
2010	112 (7.4)
2011	82 (5.4)
2012	106 (7.0)
2013	165 (10.8)
2014	178 (11.7)
2015	349 (23.0)
ART regimen (*n* = 1288)	
TDF/3TC/EFV	529 (41.1)
AZT/3TC/NVP	746 (57.9)
Others	13 (1.0)
Current WHO stage	
Stage I	348 (22.9)
Stage II	680 (44.7)
Stage II	313 (20.6)
Stage IV	180 (11.8)
Daily dose of cotrimoxazole	
No	749 (49.2)
Yes	772 (50.8)

ART: antiretroviral therapy; AZT: zidovudine; EFV: efavirenz; NVP: nevirapine; 3TC: lamivudine; WHO: World Health Organization.

**Table 3:** Prevalence of malaria parasite among HIV infected participants in relation to sociodemographic characteristics (age and gender) and density count among study participants attending two selected public ART clinics in Kano, Nigeria (*n* = 1521).

Variables	Malaria positive *n* (%)	Malaria negative *n* (%)	Chi-square test statistic	*p* value
RTD MP parasite species *Pf* and *Pv*	250 (16.4)	1271 (83.7)	—	—
Prevalence of malaria and HIV	386 (25.4)	1135 (74.6)	—	—
BSM MP parasite species *Pf*	383 (25.2)	—	—	—
BSM MP parasite species *Pf* and *Pv*	3 (0.2)	—	—	—
Overall *Pf* specie prevalence	383 (99.2)	—	—	—
Mixed infection prevalence	3 (0.8)	—	—	—
Age group (years)				
Less than 30 (*n* = 331)	92 (27.8)	239 (72.2)	6.499	0.165
30–39 (*n* = 596)	141 (23.7)	455 (76.3)		
40–49 (*n* = 377)	88 (23.3)	289 (76.7)		
50–59 (*n* = 161)	45 (28.0)	116 (72.0)		
60 and above (*n* = 56)	20 (35.7)	36 (64.3)		
Gender				
Male (*n* = 447)	114 (25.5)	333 (74.5)	0.005	0.942
Female (*n* = 1074)	272 (25.3)	802 (74.7)		
Malaria density count				
Malaria density count mean ± SD cells/*μ*l	265 ± 31.8	—	—	—
Malaria density range cells/*μ*l	20–2500			

BSM: blood smear microscopy; MP: malaria parasites; NS: no significant difference; *n*: number examined; *Pf: Plasmodium falciparum*; *Pv: Plasmodium vivax*; RTD: rapid diagnosis test; SD: standard deviation. ^*∗*^Significant at *p* < 0.05.

**Table 4:** Comparison of hematological parameters of HIV only and HIV and malaria dual infected participants attending two selected public ART clinics in Kano, Nigeria (*n* = 1521).

Variable	HIV+ & malaria+ (*n* = 386)	HIV+ only (*n* = 1135)	*t*-test	*p* value
Hb (g/dl)	10.95 ± 2.26	11.64 ± 1.85	5.42	0.001^*∗*^
PCV (%)	33.00 ± 6.21	35.42 ± 5.00	6.93	0.001^*∗*^
WBC (×10^9/L^)	6.13 ± 2.87	5.95 ± 2.22	−0.737	0.461
Platelet (×10^9/L^)	263 ± 18.8	261 ± 18.2	0.022	0.983
RBC (×10^12/L^)	3.79 ± 0.73	3.95 ± 0.70	3.77	0.001^*∗*^
MCV (fl)	87.78 ± 11.17	91.13 ± 10.99	5.16	0.001^*∗*^
MCHC (g/dl)	33.12 ± 2.57	32.81 ± 2.46	−2.10	0.036^*∗*^
MCH (pg)	29.19 ± 4.71	30.02 ± 4.58	3.05	0.002^*∗*^
Neutrophil (%)	50.56 ± 13.69	48.73 ± 12.78	−2.37	0.017^*∗*^
Lymphocyte (%)	39.00 ± 12.77	42.11 ± 11.10	−3.53	0.001^*∗*^
Mixed (%)	9.93 ± 6.02	9.14 ± 5.85	−2.27	0.024^*∗*^
Abs neut (×10^9/L^)	4.42 ± 0.23	4.20 ± 0.21	−1.69	0.910
Abs lymph (×10^9/L^)	2.29 ± 1.02	3.50 ± 0.18	3.04	0.002^*∗*^
Abs mixed (×10^9/L^)	1.91 ± 0.12	1.80 ± 0.11	−1.86	0.630
RDW-SD (%)	48.83 ± 6.29	49.79 ± 6.83	−2.40	0.016^*∗*^
PDW	10.80 ± 0.08	10.70 ± 0.08	−1.34	0.180

Abs neut: absolute neutrophil; abs lymph: absolute lymphocyte count; Hb: hemoglobin; MCV: mean cell volume; MCHC: mean cell hemoglobin concentration; PCV: packed cell volume; PDW: platelet distribution width; SD: standard deviation; WBC: white blood cell; RBC: red blood cell; RDW: red cell distribution width. ^*∗*^Significant at *p* < 0.05.

**Table 5:** Association between malaria parasite density count and hematological parameters of HIV and malaria dual infected participants attending two selected public ART clinics in Kano, Nigeria (*n* = 386).

Variables	Mean ± SD	*R * ^2^	Unstandardized *β*	95% CI for *β*	*t*-statistic	*p* value
Hb (g/dl)	10.95 ± 2.25	0.019	−0.980	−1.685–−0.276	−2.738	0.006^*∗*^
RBC (×10^12/L^)	3.79 ± 0.73	0.012	−0.252	−0.479–−0.025	−2.181	0.030^*∗*^
MCV (fl)	87.78 ± 11.18	0.002	1.423	−2.097–4.942	0.795	0.427
MCH (pg)	29.19 ± 4.71	0.005	−1.017	−2.497–0.462	−1.352	0.177
MCHC (g/dl)	33.12 ± 2.57	0.048	−1.756	−2.545–−0.967	−4.376	0.001^*∗*^
Neutrophils (%)	50.56 ± 13.69	0.026	6.955	2.696–11.213	3.211	0.001^*∗*^
Lymphocytes (%)	39.58 ± 12.77	0.020	−5.677	−9.660–−1.693	−2.802	0.005^*∗*^
Mixed (%)	9.93 ± 6.02	0.005	−1.390	−3.281–0.501	−1.445	0.149
Abs lymph (×10^9/L^)	2.29 ± 1.02	0.017	−0.083	−0.145–−0.020	−2.596	0.010^*∗*^
RDW	48.83 ± 6.29	0.016	0.021	0.004–0.038	2.483	0.013^*∗*^

Abs lymph: absolute lymphocyte count; Hb: hemoglobin; MCV: mean cell volume; MCHC: mean cell hemoglobin concentration; PCV: packed cell volume; RDW: red cell distribution width; SD: standard deviation, MCH: mean cell hemoglobin. ^*∗*^Significant at *p* < 0.05.

## Data Availability

Data associated with this study are available in this paper in the form of tables, and any other data needed are available on reasonable request through the corresponding author.
